# Identification of individual components of a commercial wheat germ acid phosphatase preparation

**DOI:** 10.1371/journal.pone.0248717

**Published:** 2021-03-22

**Authors:** Veronica R. Moorman, Alexandra M. Brayton

**Affiliations:** Department of Chemistry and Biochemistry, Kettering University, Flint, Michigan, United States of America; CSIR - Institute of Himalayan Bioresource Technology, INDIA

## Abstract

Wheat germ acid phosphatase (WGAP) is a commercial preparation of partially purified protein commonly used in laboratory settings for non-specific enzymatic dephosphorylation. It is known that these preparations contain multiple phosphatase isozymes and are still relatively crude. This study therefore aimed to identify the protein components of a commercial preparation of wheat germ acid phosphatase using mass spectroscopy and comparative genomics. After one post-purchase purification step, the most prevalent fifteen proteins in the mixture included heat shock proteins, beta-amylases, glucoseribitol dehydrogenases, enolases, and an aminopeptidase. While not among the most abundant components, eight unique dephosphorylation enzymes were also present including three purple acid phosphatases. Furthermore, it is shown that some of these correspond to previously isolated isozymes; one of which has been also previously shown by transcriptome data to be overexpressed in wheat seeds. In summary, this study identified the major components of WGAP including phosphatases and hypothesizes the most active components towards a better understanding of this commonly used laboratory tool.

## Introduction

Phosphatases catalyze the hydrolysis of phosphoric-monoester compounds to produce a free inorganic phosphate and an alcohol. While the classification of phosphatases with specificity is based on the substrates they act upon (such as phytases), promiscuous phosphatases are classified as either acid or alkaline based on the environments that they best operate in and are further classified by their reaction mechanism [1]. Purple acid phosphatases (PAPs), for example, are metalloenzymes that appear purple or violet in their oxidized form.

Commercial wheat germ acid phosphatase (WGAP) is extracted from the germ/embryo of wheat plants and is commonly used *in vitro* in many biochemistry laboratories. Specifically, its availability and inexpensive cost have historically made it a common choice for many projects which require a broad specificity phosphatase, including those in education and research (see, for example, references [2–12]). While the name wheat germ acid phosphatase implies a singular protein, it typically refers to a commercial preparation of partially purified protein which is known to contain multiple proteins [13–18]. Knowledge of the individual component identities would allow scientists using commercial WGAP to have a better molecular understanding of its mechanism of action and therefore determine whether it is an appropriate phosphatase for their purposes. Despite numerous published laboratory activities to purify these mixtures [19–22], the precise active phosphatase of this mixture, however, has not previously been identified.

Since the genome of common wheat (*Triticum aestivum*) is hexaploid (AABBDD) and is presumed to have been formed through the hybridization of the diploid *Aegilops tauschii* (DD) with the tetraploid *Triticum dicoccoides* (AABB) who in turn was formed through hybridization of *Triticum urartu* (AA) with an unknown BB donor [23], there are many paralogous genes and alleles present, making gene and protein identification historically difficult. The recent availability of the complete wheat genome [24, 25] coupled with the increased use of protein identification by mass spectroscopy [26] has, however, made this type of deconvolution possible. Using trypsin degradation and subsequent mass spectroscopy to identify peptide fragments from a commercial preparation of acid phosphatase from wheat germ, the phosphatase components were identified and described here.

## Materials and methods

Acid Phosphatase from Wheat Germ (Sigma-Aldrich P3627) in 41 mM sodium citrate (pH 4.8) was centrifuged, filtered, and subjected to size exclusion chromatography using a Superdex 75 Increase 10/300 GL 10 × 300 mm column at a flow rate of 0.7 mL/min. 0.5 mL fractions were collected between 10 and 19 minutes, during which time the signal was detected at 280 nm by an in-line diode array detector. A small portion of each fraction was run on an SDS-PAGE to look for inhomogeneity. Additionally, activity was determined by absorbance at 410 nM after incubation of a small amount with 10 mM *p*-nitrophenyl phosphate in 41 mM sodium citrate (pH 4.8) for 15 minutes and quenched with sodium hydroxide. These fractions corresponded to 14.2–16.3 minutes of elution, which, when detected at 280 nm, showed three visible peaks corresponding to molecular weights of 46733, 27863, and 19160 g/mol, when determined using GE Healthcare’s Low Molecular Weight Gel Filtration Calibration Kit. Fractions with phosphatase activity were pooled and concentrated 600-fold.

Protein identification mass spectroscopy (LC-MS/MS) and analysis were performed by MS Bioworks LLC (Ann Arbor, MI). Briefly, the sample was hydrolyzed with trypsin and run over tandem mass spectroscopy. MS data were searched using Mascot (Matrix Science, London, UK; version 2.6.2) against a FASTA file of the annotated genome of *Triticum aestivum* (see above) with the following parameters: Fragment Tolerance- 0.020 Da; Parent Tolerance- 10.0 PPM; Fixed Modifications- Carbamidomethyl; Variable Modifications- Gln->pyro-Glu of the n-terminus, deamidated of asparagine and glutamine, oxidation of methionine, acetyl of the N-terminus of lysine, and phosphorylation of serine, threonine, and tyrosine; Digestion Enzyme- strict trypsin; and Max Missed Cleavages- 2. The resulting DAT files were parsed using Scaffold (Proteome Software Inc., Portland, OR, version 4.10.0) to validate MS/MS-based protein identifications. Protein identifications were accepted if they could be established at greater than 70.0% probability to achieve an FDR less than 1.0% and contained at least two identified peptides assigned by the Protein Prophet algorithm [27]. Proteins that contained similar peptides and could not be differentiated based on MS/MS analysis alone were grouped to satisfy the principles of parsimony. The identity or predicted identity of each matching protein was determined through either the original FASTA file (identity) or through standard protein Basic Local Alignment Search Tool (BLASTp) protocols [28].

Annotated genomes were obtained from Ensembl Plants [29] and searched for the number of included proteins, phosphatases, acid phosphatases, and purple acid phosphatases. BLAST was used for similarity searching against NCBI’s non-redundant protein sequence dataset or NCBI’s Protein Data Bank protein dataset. Searching against genomic datasets, however, was conducted using a locally installed version of BLAST+ [30]. Additional information about each sequence including amino acid compositions and molecular weights was calculated using ExPASy’s ProtParam tool [31]. Isoelectric points (pI) were computationally calculated using the average across multiple methods [32]. Protein sequences were aligned with the Multiple Alignment using Fast Fourier Transform [33] implementation of the Bioinformatics Institute [34]. Alignments were visualized using images made using ExPASy’s BoxShade server [31]. Transcriptomic data was found in the searchable database WheatExp [35] which compiles six transcriptional studies on wheat [36–41]. Each sequence of interest was BLASTed (tBLASTn) [30] against the wheat transcriptome to find the most similar sequences (cut off at an E-value of 1x10^-10^).

## Results and discussion

The annotated genomes from four wheat species (*Triticum aestivum*, *Triticum dicoccoides*, *Triticum urartu*, and *Aegilops tauschii*) were investigated in the present study. The total number of proteins identified for each annotated genome as well as the number of identified phosphatases, acid phosphatases, and purple acid phosphatases (PAPs) are compiled in Table 1. Interestingly, while the total number of proteins and phosphatases did not seem to match what would be expected from the ploidy of each species, the number of acid phosphatases and purple acid phosphatases did follow the expected ratio, indicating that acid phosphatases may be retained at a greater rate than other phosphatases.

**Table 1:** Numbers of total proteins, phosphatases, and purple acid phosphatases (PAPs) identified in the annotated genomes.

			Proteins in annotated genome		
Species		Ploidy	Total	Phosphatases	Acid Phosphatases (PAPs)
*Triticum aestivum*	Common wheat	AABBDD	133,346	355	123 (123)
*Triticum dicoccoides*	Emmer or hulled wheat	AABB	295,286	381	56 (56)
*Triticum urartu*	Red wild einkorn wheat	AA	33,483	206	35 (27)
*Aegilops tauschii*	Goatgrass or rough-spike hard grass	DD	258,680	291	35 (35)

Three hundred and thirty-seven *Triticum aestivum* proteins were identified in the dephosphorylating fractions of the commercial preparation of wheat germ acid phosphatase. These proteins fell into one of seven broad biological function categories: metabolism (149), defense/detox (73), embryo/seed-specific (51), regulation (23), macromolecule production (14), signaling/movement (9), or unknown (18). Each of these genes was also categorized by their chemical function: hydrolase (94), oxidoreductase (48), lyase (15), transferase (24), isomerase (16), ligase (4), translocase (4), non-enzyme (117), and unknown chemical function (15). As assessed by total spectrum counts, the most prevalent fifteen proteins in the mixture included five heat shock proteins, three beta-amylases, three glucose/ribitol dehydrogenases, three enolases, and one aminopeptidase. Therefore, despite its name and known activity, the composition of commercial wheat germ acid phosphatase is not primarily phosphatase, even after an additional purification step.

Of these 337 wheat proteins identified, however, eight were able to be identified as having dephosphorylation activity and are therefore listed in Table 2. Additional similar proteins from the original dataset which were found to have less than three percent differences in sequence or be identical with just a leading sequence to those original hits. These hits had total spectrum counts of approximately 10–20% of those from the top fifteen hits discussed above, showing that they are likely present in a much lower amount than those top proteins.

**Table 2:** Common wheat dephosphorylation genes identified in WGAP mixture.

	Size (kDa)	pI	Identity	EC #	Biological Function
TraesCS3A02G103900.1	52	6.4	Nucleotide pyrophosphatase/ phosphodiesterase ^a^	3.6.1.9	Metabolism
TraesCS4B02G161300.1	29	5.3	Inositol-1-monophosphatase	3.1.3.25	Signaling/Movement
TraesCS5D02G335100.1	60	6.0	Purple acid phosphatase	3.1.3.2	Embryo/Seed-Specific
TraesCS5D02G335100.2^b^					
TraesCS4A02G163700.1	27	4.7	Inositol-1-monophosphatase	3.1.3.25	Signaling/Movement
TraesCS6B02G148200.1	32	8.1	Protein phosphatase 2C 10 ^a^	3.1.3.16	Defense/Detox
TraesCS6D02G109800.2^b^					
TraesCS7B02G434100.1	55	5.5	Purple acid phosphatase	3.1.3.2	Embryo/Seed-Specific
TraesCS7A02G517900.1^b^					
TraesCS7D02G507900.1^b^					
TraesCS4D02G231400.1	38	4.7	Purple acid phosphatase	3.1.3.2	Embryo/Seed-Specific
TraesCS3B02G149100.1	92	5.1	Phospholipase D	3.1.4.4	Metabolism
TraesCS3A02G130000.1^b^					
TraesCS3D02G130900.1^b^					

^a^ Identified by BLAST only.

^b^ Identified as similar by Scaffold.

Notably, each of the eight genes identified here shows strong sequence identity (BLAST E-values of at most 1x10^-5^) to at least one of genes in each of the four wheat species’ annotated genomes discussed previously. In some cases there was strong sequence identity to dozens of proteins, indicating that these genes have likely multiple paralogs, orthologs, and/or alleles. Indeed, these homologs extend beyond wheat species, as each one also shares sequence high identity with proteins in other plant species as well.

**Fig 1:**
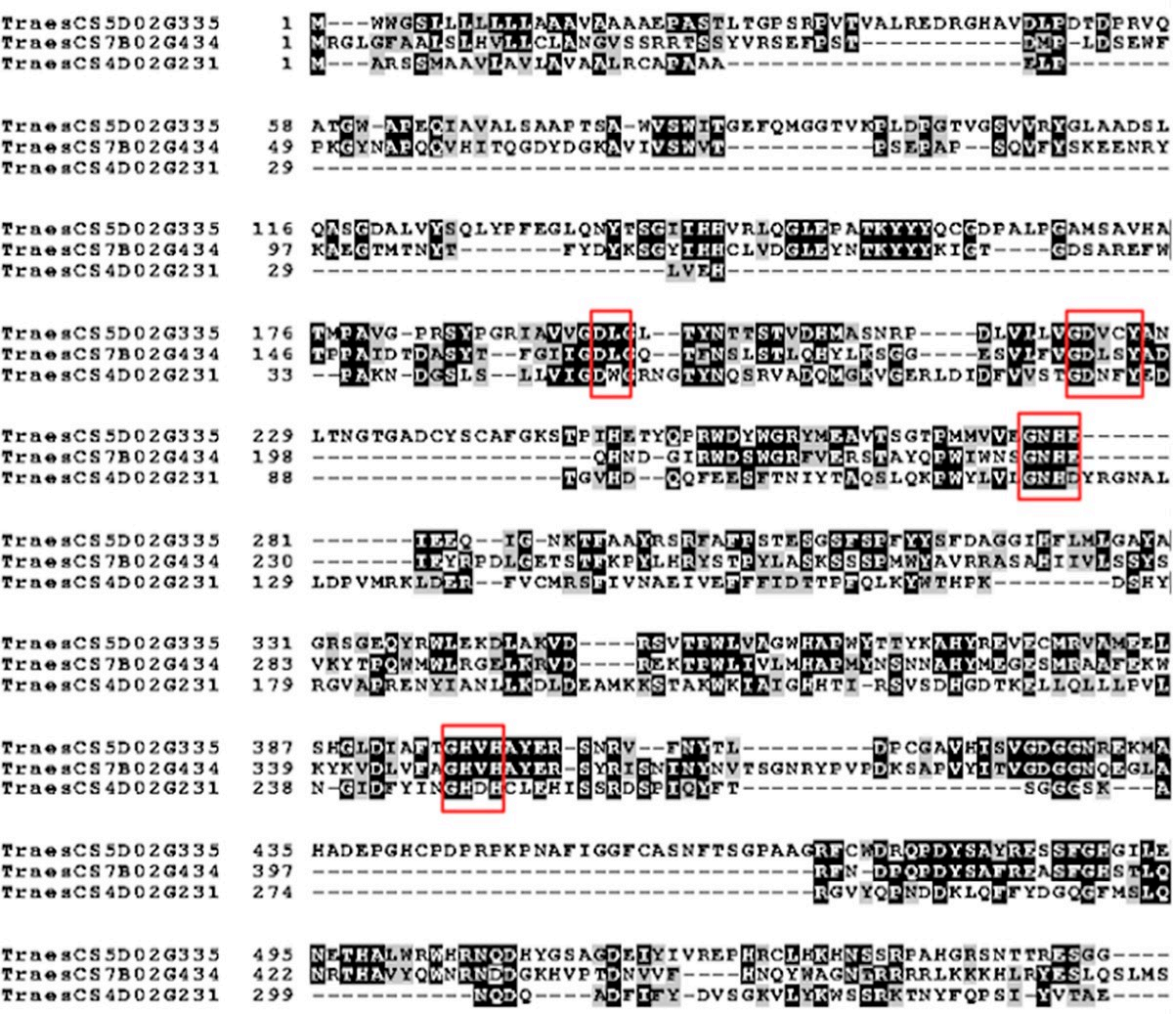
MAFFT sequence alignment of the three unique purple acid phosphatases identified in commercial WGAP. Black shading indicates identical residues in that position, while grey indicates conservative changes. Red boxes identify known purple acid phosphatase sequence motifs.

As seen in Table 1, three of the identified phosphatases (TraesCS5D02G335100, TraesCS7B02G434100, and TraesCS4D02G231400) are purple acid phosphatases (PAPs), which are known to be important in plants for phosphorus assimilation in developing plants [42, 43] and are therefore classified as important for embryos and/or being seed-specific. One of them (TraesCS4D02G231400) is expected to be a member of the small PAP class comprising of monomeric PAPs of approximately 35 kDa, while the other two (TraesCS5D02G335100 and TraesCS7B02G434100) are expected to be of the large PAP class which are homodimers with each chain being approximately 55 kDa [42]. Despite both types being present in plants and animals, only the small class has been well-characterized in animals while only the larger class has been well-characterized in plants [44]. Aligning all three of these sequences, as can be seen in Fig 1, shows not only the commonly conserved purple acid phosphatase hallmarks including the metal binding residues (DXG, GDXXY, GNH(D/E), GHXH) [44, 45] across all three but also clearly demonstrates that the larger proteins are quite similar to each other, despite TraesCS5D02G335100 containing several insertions. Specifically, TraesCS7B02G434100 and TraesCS4D02G231400 have 33.8% and 34.1% sequence identity to TraesCS5D02G335100, respectively, and is illustrated in Fig 1.

In addition to containing characteristic sequence motifs, PAPs are known to bind two three-dimensionally close metal ions including one at a conserved tyrosine which results in their purple color, have their phosphatase activity uninhibited by L-tartrate, and are highly glycosylated [44]. While one PAP in soybean seedlings was found to have phytase activity [46], until recently this was thought to be uncommon amongst plant PAPs. Wheat phytase has recently, however, been seen to have activity against pNPP (WGAP’s canonical substrate), while WGAP has activity against phytate (phytase’s defining substrate) indicating that there may be some overlap between two enzyme classifications [47]. The annotated genomes of wheat germ, however, have no mention of any phytases (although some phosphatases may be able to hydrolyze phytate).

In the decades since partially purified wheat germ acid phosphatase preparations were first described [48, 49], multiple groups have purified and characterized various isozymes [13, 15–18, 50–55]. Here, matching the published sequences of those isolated phosphatases with the ones identified by mass spectrometry was attempted to help reconcile the different isozymes identified as being WGAP. The small purple acid phosphatase, TraesCS4D02G231400, matched the partial sequence previously identified as isozyme B-4 [18]. The smaller of the large PAP was unable to be identified from the literature and thus may not have ever been isolated. The largest purple acid phosphatase (TraesCS5D02G335100), however, matched the known wheat germ acid phosphatase components AX298209 [55] as well as the a1 isoform [52] of *Triticum aestivum*’s purple acid phytase TaPAPhy [51]. One of the other identified dephosphorylation enzymes was also able to be matched with a previously identified protein. The identified phospholipase D (TraesCS3B02G149100) compared closely in sequence with *Triticum aestivum*’s TaPLDα that was identified in 2014 and has high sequence similarities to proteins in multiple plant species [56].

While identifying isozyme components of the crude mixture is useful for some biotechnological applications, biological relevance may also be of interest, as variables like age at harvest or environment can have a large effect on phosphatase requirements in growing plants. Toward this goal, the identified phosphatases were also found in transcriptome data compiled in WheatExp [35]. For each phosphatase enzyme examined, multiple protein IDs were identified in the WheatExp database indicating that the proteins identified by mass spectrometry may have multiple paralogs in wheat. Of particular note were the results from the purple acid phosphatase genes. TraesCS5D02G335100 had a match (Traes_5BL_6E019E8E7.1) which had much higher expression in grain than in leaf, root, spike, and stem which increases over day 75 to 85 [36] and higher expression in the inner pericarp compared with the endosperm (although the germ itself was not tested) [38] with much higher expression levels in the endosperm at day 20 than day 10 with the majority of the expression in the aleurone of the endosperm [37]. This was the clearest pattern of differential expression, but the other phosphatases had some interesting trends as well. TraesCS7B02G434100 had a corresponding WheatExp ID (Traes_7BL_1AB53A2AF.1) with the highest expression levels in grain (with the highest levels at day 75 compared with day 71 and 85) [36] with much higher expression in the endosperm than in the pericarp [38]. TraesCS4D02G231400’s matches (including Traes_4BL_8E6854176.1) were fairly evenly expressed across the parts of the plant, but their expression levels decreased substantially in the grain by day 85 [36]. Comparing these three genes, TraesCS5D02G335100 had the highest expression levels. Interestingly, despite each of the genes identified via mass spectroscopy having multiple matches in the WheatExp database, each of these matches did not show a similar profile. This indicates that paralogs do not necessarily show similar expression patterns, which is not unexpected in polypoids. Additionally, as there were some clear differences in expression levels in the grain at different times of germination, the timing of germ harvest will likely greatly affect phosphatase activity (and potentially even which phosphatase is most present), something that was also noted decades ago [57]. Finally, as these phosphatases have high expression in the endosperm, the germ may not be the most efficient location for harvesting. These differences may be particularly interesting to those who wish to optimize WGAP expression or to those who may be concerned about differential activity amongst commercial preparations.

In conclusion, the individual phosphatase enzymes collectively known as wheat germ acid phosphatase have now been identified, as have other major components in a commercial preparation. Multiple of the phosphatase sequences identified here match with those of proteins isolated and/or characterized previously, helping to reconcile previous identifications. This information will allow for subsequent work on these individual proteins to better understand their exact role in commercial mixtures and will allow scientists using WGAP as a generic phosphatase to have a better molecular understanding of how it operates.

## Supporting information

S1 File Proteins identified by mass spectroscopy in wheat germ acid phosphatase mixture. (XLSX)
